# Effects of Multiple Sessions of Cathodal Priming and Anodal HD-tDCS on Visuo Motor Task Plateau Learning and Retention

**DOI:** 10.3390/brainsci10110875

**Published:** 2020-11-19

**Authors:** Pierre Besson, Makii Muthalib, Christophe De Vassoigne, Jonh Rothwell, Stephane Perrey

**Affiliations:** 1 EuroMov Digital Health in Motion, Univ Montpellier, IMT Mines Ales, 34090 Montpellier, France; pierre.besson@umontpellier.fr (P.B.); makii.muthalib@gmail.com (M.M.); ch.de.vassoigne@gmail.com (C.D.V.); 2 SilverLine Research, 4127 Brisbane, Australia; 3 Institute of Neurology, University College London, London WC1N 3BG, UK; j.rothwell@ucl.ac.uk

**Keywords:** transcranial direct current stimulation (tDCS), motor performance, priming tDCS, cathodal, multiple sessions, motor learning, neuroplasticity

## Abstract

A single session of priming cathodal transcranial direct current stimulation (tDCS) prior to anodal tDCS (c-a-tDCS) allows cumulative effects on motor learning and retention. However, the impact of multiple sessions of c-a-tDCS priming on learning and retention remains unclear. Here, we tested whether multiple sessions of c-a-tDCS (over 3 consecutive days) applied over the left sensorimotor cortex can further enhance motor learning and retention of an already learned visuo-motor task as compared to anodal tDCS (a-tDCS) or sham. In a between group and randomized double-blind sham-controlled study design, 25 participants separated in 3 independent groups underwent 2 days of baseline training without tDCS followed by 3-days of training with both online and offline tDCS, and two retention tests (1 and 14 days later). Each training block consisted of five trials of a 60 s circular-tracing task intersected by 60 s rest, and performance was assessed in terms of speed–accuracy trade-off represented notably by an index of performance (IP). The main findings of this exploratory study were that multiple sessions of c-a-tDCS significantly further enhanced IP above baseline training levels over the 3 training days that were maintained over the 2 retention days, but these learning and retention performance changes were not significantly different from the sham group. Subtle differences in the changes in speed–accuracy trade-off (components of IP) between c-a-tDCS (maintenance of accuracy over increasing speed) and a-tDCS (increasing speed over maintenance of accuracy) provide preliminary insights to a mechanistic modulation of motor performance with priming and polarity of tDCS.

## 1. Introduction

Transcranial direct current stimulation (tDCS) is a noninvasive neuromodulation technique that can increase or decrease cortical excitability depending on the polarity of the induced electric field [1]. Anodal tDCS (a-tDCS) of the primary motor cortex (M1) has generally been shown to enhance motor performance and learning, but this depends on the specific motor task utilized [2], as well as tDCS parameters (electrode position [3]; current intensity/density [4]) and the timing of application [5,6]. However, even with strict control of these considerations, intra- and inter-individual variability of responses to tDCS have been reported in several studies [7,8]. Although anatomical differences between subjects will always be a major factor influencing tDCS responses, one way to enhance tDCS responses is to design new tDCS protocols where personalization of stimulation parameters is the ultimate goal [9]. Regarding the tDCS setup, high-definition (HD)-tDCS montage can be one solution to improve optimization of the technique due to the expected focality of the induced-current [10,11] and the persistence of the after-effects on cortical excitability [12,13].

For either motor or cognitive tasks, concurrent (online) application of a-tDCS and task training is a potential way to enhance the performance and learning [14,15]. Motor learning [16] is typically defined as practice- or experience-induced acquisition of either fine motor skills from increased accuracy and reduced performance variability (speed–accuracy trade-off phenomenon) or gross skilled motor performance permitting functions as jumping, walking, maintaining a body balance, etc. Most studies (e.g., [4,5]) have tested the efficacy of tDCS coupled with learning of fine motor skills. The greater facilitative effect of concurrent a-tDCS on motor performance/learning is thought to be due to enhanced synaptic efficacy in the simultaneously engaged neural network through a “gating” mechanism [17]. The seminal work of Antal et al. [18] has shown that the excitability enhancement of M1 induced by a-tDCS improved performance in the early phase of learning in a visuo-motor coordination task compared to sham. Offline a-tDCS (i.e., tDCS before the task) has been suggested to limit motor performance/learning compared to online a-tDCS due to homeostatic metaplastic mechanisms based on the Bienenstock–Cooper–Munro theory claiming a “sliding threshold” for bidirectional synaptic plasticity [17]. Accordingly, a-tDCS, which increases the likelihood of long-term potentiation (LTP)-like plasticity, would increase the modification threshold for LTP during the subsequent motor task and thus adversely affect motor performance/learning [14]. Simultaneous application of tDCS and training appears a requirement to promote offline gains in favour of retention process [5]. Our recent functional near infrared spectroscopy neuroimaging study [6] observed that although online a-HD-tDCS showed reduced sensorimotor cortex activation to offline a-HD-tDCS relative to when the motor task is performed. However, after a 30 min delay in motor task performance, sensorimotor cortex activation was similarly increased for both online and offline compared to sham. Altogether, in healthy adults, a meta-analysis [19] concluded that multiple sessions of a-tDCS are more efficacious than a single session for enhancing both motor learning and retention, due to combined incremental online and offline skill gains.

The sequence and timing of the tDCS polarity are two factors that can also be manipulated to enhance motor performance and learning with regard to the homeostatic metaplasticity phenomenon [20]. Sub-threshold neuronal membrane depolarization induced by a-tDCS has an intensity- and time-dependent effect to strengthen synaptic efficacy [21]. Reducing corticospinal excitability with priming cathodal tDCS (c-tDCS) before a-tDCS (c-a-tDCS) and motor task training can influence homeostatic metaplastic mechanisms as well [22,23]. Applying priming c-tDCS followed 10-min later by concurrent a-tDCS and motor task training appears promising to induce significantly greater enhancement in acquisition [23] and retention of motor skills two weeks later [22] as compared to sham and training with concurrent a-tDCS. However, to the best of our knowledge, no study has compared multiple sessions of c-tDCS priming and a-tDCS (c-a-tDCS) to further enhance plateau learning and retention of an already learned motor skill. Herein we aimed to investigate the beneficial effect of a new tDCS protocol exploiting c-tDCS priming on online gains, offline gains and long-term retention after multiple days of motor practice. For that purpose, 3–5 training days are regularly used [2,5,24,25]. We adopted a 3-day training phase as carried out in the studies of Saucedo Marquez et al. [2] regarding fine motor skills, or Kumari et al. [24] regarding gross motor skills.

Therefore, the aim of this exploratory study was to determine if multiple sessions (over 3 consecutive days) of c-a-tDCS can further enhance motor learning and retention of an already learned visuo-motor task. Based on the aforementioned studies, we hypothesized that c-a-tDCS would induce a greater improvement in learning and retention compared to a-tDCS or sham.

## 2. Materials and Methods

### 2.1. Participants

Twenty-five healthy adults (9 females, 19–45 years old, mean age ± SD: 31.0 ± 9.9) volunteered to participate in the study. All participants gave written informed consent prior to participation in the study according to the Declaration of Helsinki. All procedures were approved by the local Ethics Committee (IRB-EM 17-01B, EuroMov-Montpellier). The laterality index for right handers (*n* = 21 with a-tDCS = 6, c-a-tDCS = 8 and sham = 7) and left handers (*n* = 4 with a-tDCS = 3 and c-a-tDCS = 1) assessed with the Edinburg handedness inventory [26] was 75 ± 23 and −70 ± 33, respectively. All participants had no history of neurology or physical disorders or any upper extremity muscle or joint injuries. The respect of safety recommendations (e.g., current duration, current density, charge density) associated with the use of tDCS was strictly followed [27].

### 2.2. Study Design and Protocol

**Figure 1:**
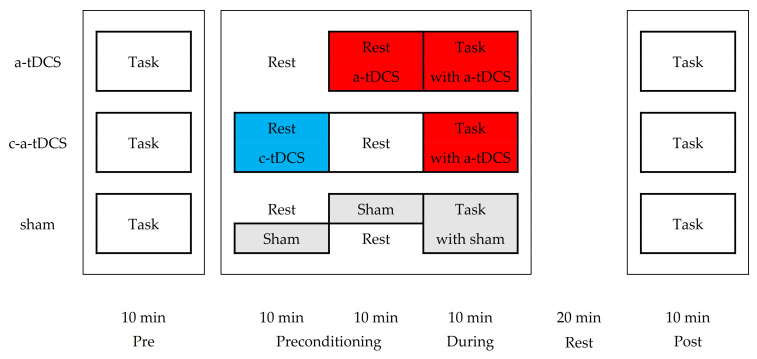
Schematic representation of a training day (3 blocks) for the 3 groups. The subject performed the visuo-motor task (5 trials) at three epochs interspersed by 20 min of preconditioning or delay. Pre and post times are without use of transcranial direct current stimulation (tDCS) while during depends on the specific tDCS conditions of the 3 groups exploiting different polarities (anodal: red; cathodal: blue; sham: grey) in the preconditioning phase.

This study is a part of Dr. Pierre Besson’s PhD thesis. In a double-blind sham-controlled study [28], the 25 participants were randomly distributed into 3 groups: anodal anodal-task (a-tDCS, *n* = 9, 3 females, age 31.0 ± 8.9); cathodal priming/anodal-task (c-a-tDCS, *n* = 9, 4 females, age 31.7 ± 12.0); sham (*n* = 7, 3 females, age 30.1 ± 8.9). For sham, 3 participants underwent a-tDCS and 4 underwent c-a-tDCS. All participants were required to undertake 6 testing days (5 successive days and one day 2 weeks later). For the baseline (day 0) and the 2 retention testing days (day 4 and day 18), no tDCS was applied and only the tracing-motor task consisting of 1 block (B) of 5 trials (1 min task interspersed by 1-min rest, total 10 min duration) was performed. Days 1, 2 and 3 were training days and included either sham or real tDCS. Figure 1 presents the schematic of the experimental design for a training day. Each training day was comprised of 3 blocks of 5 trials: pre-tDCS block, tDCS-block and post-tDCS block. In the pre-tDCS block, no tDCS was applied to all groups during the tracing-motor task. In the tDCS-block, the specific tDCS parameters were set and concurrent tDCS and tracing-motor task training were undertaken; a-tDCS priming (10 min) was next to online a-tDCS task (10 min) while c-tDCS priming (10 min) was interspersed by 10 min of rest before the online a-tDCS task (10 min) (Figure 1). In the post-tDCS block, the tracing-motor task was performed again with no tDCS after 20 min rest to assess within-day offline effects. Subjects were informed to perform the tracing-motor task as fast as possible while maintaining accuracy.

All participants and one experimenter (C.D.V.) performing the tDCS applications/assessment were blind to the tDCS settings. Although tDCS is well tolerated by participants [29], a questionnaire containing rating scales of 11 unpleasant sensations compared to resting state (i.e., sitting quietly without tDCS electrodes over the head) was filled out after each stimulation sequence. This questionnaire was based on the tDCS safety guidelines proposed by Poreisz et al. [30].

### 2.3. Transcranial Direct Current Stimulation

A Startstim 8 tDCS system (Neuroelectrics^®^, Barcelona, Spain) was used to deliver constant direct currents to the left (right handers, *n* = 21) or right (left handers, *n* = 4) M1 via a 4 × 1 ring montage with HD electrodes (3.14 cm^2^) applied on the skull with electrode paste (Ten20^®^, Weaver and Company, Aurora, CO, USA). With regard to the handedness of the participant, the active electrode was placed on the scalp overlying the dominant M1 (C3 or C4) based on the 10–20 EEG system. The 4 return electrodes surrounded the anode or cathode electrode at a centre-to-centre distance of 3.5 cm. For the anode on C3, return electrodes were placed on FC1, FC5, CP1 and CP5. For the anode on C4, return electrodes were placed on FC2, FC6, CP2 and CP4. To ensure consistency of electrodes placement throughout the multiple training sessions, the same experimenter (C.D.V.) always marked on the scalp the site of the electrodes.

In a-tDCS conditions, constant current was delivered for either 10 min or 20 min at 2 mA with a ramp up and down phases of 30 s duration. In sham, active stimulation was applied with 30 s ramp up to 2 mA, 30 s at 2 mA and 30 s ramp down (1.5 min active stimulation, [28]). For the c-a-tDCS group, c-tDCS was applied for 10 min with 30 s ramp up/down, then after a 10 min rest, a-tDCS was applied for 10 min with 30 s ramp up/down. In all testing sessions, the impedance of all electrodes was monitored at the beginning and during each period of stimulation to maintain values under 5 kΩ.

### 2.4. Visuo-Motor Task

**Figure 2:**
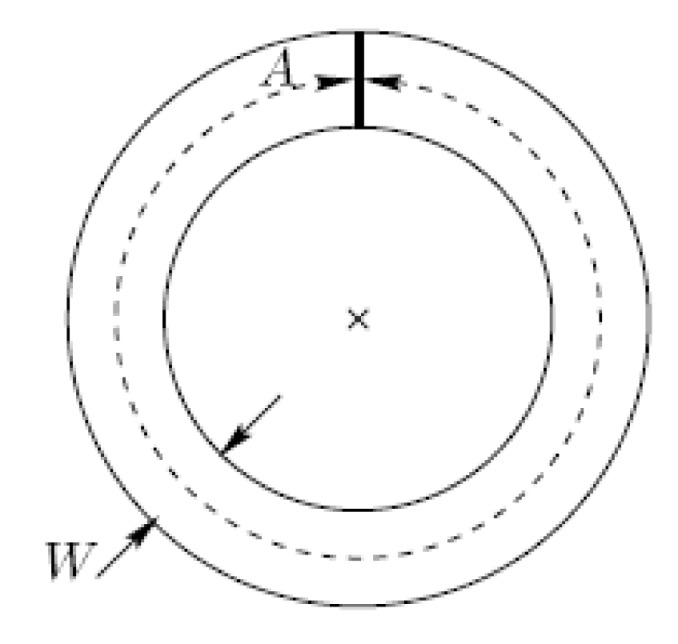
Representation of the circular-tracing task. A is the perimeter (dotted line) of the circle’s centre (x) and W stands for the path width (continuous lines). From Accot and Zhai [31].

The visuo-motor task was a computerized version of the circular tunnel task shown to be highly reliable over testing days [31]. Subjects were required to do circular traces as quickly as possible using a hand stylus within the boundaries of a circle of an 80 cm length and targeting the centre of 0.8 cm width (accuracy purpose) from 12.3 to 13.1 cm (see Figure 2). The index of difficulty (ID) defined by the length of circle (A) divided by the channel’s width (W) was set to 100 (i.e., 80/0.8) [32]. The line tracing was recorded with a computerized tablet Wacom Intuos (gd1218U, Saitama, Japan) at the sampling frequency of 100 Hz. For data acquisition, a homemade script was created using MATLAB^®^ (version R2012b—MathWorks, Natick, MA, USA).

### 2.5. Data Analysis

We defined an index of performance (IP, arbitrary unit) for the task based on previous related studies [32,33] as follows:

IP = TED60/WVT60(1)

where TED60 represents the total Euclidean distance achieved during the 60-s task and WVT60 represents the width of the virtual circular tunnel, including all the trajectories of the subject during the 60-s task.

To calculate IP, we developed a Matlab script taking in input raw data from the Wacom Intuos tablet. The first step in pre-processing raw data was calibration. For that step, we used a controlled data set and transformed the pixel indexes (X and Y positions) into Euclidean distance (in mm) from the centre of the circular tunnel. The second step consisted in re-sampling the data to obtain a fixed sampling period at 100 Hz; the interp1 function of Matlab with the “pchip” method of interpolation was used. IP was computed from the pre-treated data where TED60 was calculated by summing the Euclidean distances between 2 consecutive points for all points acquired during the motor task. WVT60 was calculated as the difference between the distance from the farthest point to the centre and the distance from the nearest point to the centre for all points. With respect to the purpose of the study, IP values and its determinants (speed and accuracy) were assessed by block of 5 trials. The speed was calculated with respect to the number of revolutions made during the 60 s. The error (accuracy) was assessed by the ratio of the number of samples outside the tunnel to the total number of samples recorded during the task.

### 2.6. Statistical Analysis

Values are presented as means and standard deviations except if specified. The Shapiro–Wilk test was used to examine the normal distribution of the outcomes while the sphericity assumption was tested with Mauchly’s test. All data (IP, error and speed values) were subjected to repeated-measures analysis of variance (ANOVA_RM_) with time (10 blocks normalized by the subtraction of B2 result as baseline, see below) as within-subject factor and polarity (3 groups: a-tDCS, c-a-tDCS and sham) as between-subject factor. A two-way ANOVA_RM_ was also conducted for the subjective scalp sensation related to tDCS conditions. Where appropriate, post-hoc tests using the Bonferroni correction were applied. All statistical analyses were performed using JASP software (version 0.12.1.0, JASP, 2020, Amsterdam, The Netherlands). The level of significance was set to 0.05 for all tests. Effect size (ηp^2^) values were reported for ANOVA, and effect sizes were reported with the magnitude of Hedges’ g for the simple comparisons (post hoc tests) among groups for a given time (B9, B11, B12). Hedges’ g is a variation of Cohen’s d that corrects for biases due to small sample sizes [34] and the magnitude of Hedges’ g may be interpreted using Cohen’s convention as small (0.2), medium (0.5) and large (0.8).

## 3. Results

### 3.1. Subjective Scalp Sensation

All 25 participants conducted the study to the end. ANOVA_RM_ indicated that no differences (F(2,22) = 0.0199, *p* = 0.980) were observed among the training days for the cutaneous sensation over the scalp during tDCS, indicating none of the participants were able to differentiate real tDCS from sham sessions. None of our participants reported any other tDCS application related side effects.

### 3.2. Changes in Motor Performance and Motor Learning Parameters

#### 3.2.1. Baseline Training Blocks without tDCS

Over the 2 baseline training blocks without tDCS (Day 1, B1 and Day 2, B2), there were no significant differences between groups for accuracy and IP; however, speed for a-tDCS was significantly greater than c-a-tDCS (*p* = 0.025). Speed and IP increased significantly over the baseline training blocks for c-a-tDCS (*p* = 0.019 and *p* = 0.029, respectively) and a-tDCS (*p* = 0.007 and *p* = 0.002, respectively), but not for sham. Accuracy for a-tDCS decreased significantly from B1 to B2 (*p* < 0.05); while c-a-tDCS showed a tendency (*p* = 0.051) for reduced accuracy, and sham showed no changes between blocks. Since the three groups responded differently to the baseline training, subsequent training blocks with tDCS were normalized to baseline Block 2.

#### 3.2.2. Training Blocks with tDCS

**Figure 3:**
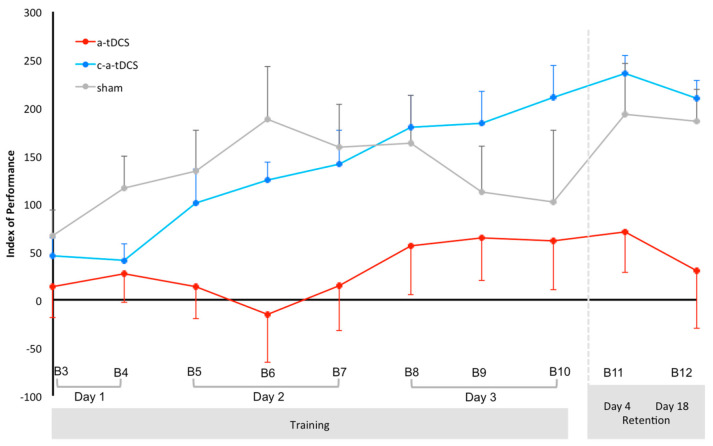
Evolution of the mean (and one SEM) index of performance over time (blocks—B) for each of the 3 groups. B3, B6 and B9 represent online tDCS. B4, B7 and B10 represent immediate offline. B5 and B8 represent delayed offline.

Figure 3 shows the evolution of normalized IP values over time (Day 1, B3 to Day 3, B10, and retention Day 4, B11 and Day 18, B12) for the 3 groups. ANOVA_RM_ for the IP indicated there were significant main effects of time (F(9198) = 5.380, *p* < 0.001, ηp^2^ = 0.196), polarity (F(2,22) = 4.730, *p* = 0.020, ηp^2^ = 0.148) and a significant time × polarity interaction (F(18,198) = 1.910, *p* = 0.017, ηp^2^ = 0.302). However post-hoc analysis failed to show any between group differences. Post hoc analysis performed on the time main effect revealed only higher IP values for c-a-tDCS at B12, B11 and B10 when compared to B3 and B4. *P*-level and effect size values are for B3 vs. B10 (*p* = 0.011, g = 1.92), B3 vs. B11 (*p* < 0.001, g = 3.21), B3 vs. B12 (*p* = 0.012, g = 2.87), and for B4 vs. B10 (*p* = 0.006, g = 2.00), B4 vs. B11 (*p* < 0.001, g = 3.39), B4 vs. B12 (*p* = 0.007, g = 3.04).

As can be seen in Figure 3, the magnitude of the difference from the start (B3) to the end of 3-days training (B10) with tDCS indicates a meaningful large increase in IP after c-a-tDCS (g = 1.92), while large increases were noted for sham (g = 0.23) and a-tDCS priming conditions (g = 0.36). In addition, the magnitude of the difference from the start (B3) to one day after the end of 3-days training (B11) indicates a meaningful large increase in IP for c-a-tDCS priming (g = 3.21), while medium increases were noted for sham (g = 1.06) and a-tDCS conditions (g = 0.49). Finally, the magnitude of the difference from the start (B3) to two weeks after the end of 3-days (B12) training indicates a meaningful large increase in IP for c-a-tDCS (g = 2.87), while medium increases was noted for sham (g = 1.39) and a-tDCS conditions (g = 0.11).

**Figure 4:**
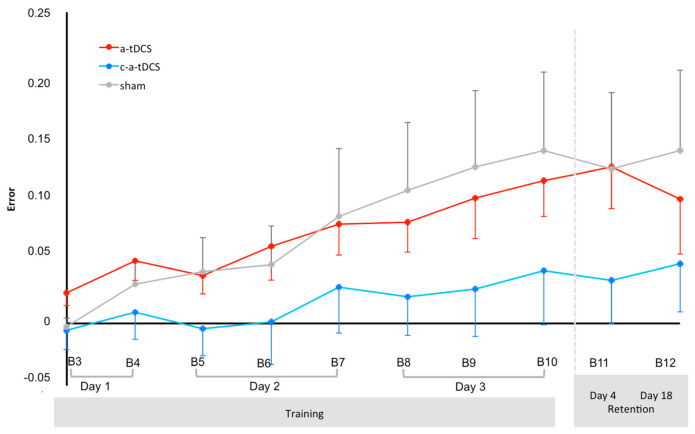
Evolution of mean (and one SEM) error over time (blocks—B) for each of the 3 groups. B3, B6 and B9 represent online tDCS. B3, B6 and B9 represent online tDCS. B4, B7 and B10 represent immediate offline. B5 and B8 represent delayed offline.

Figure 4 shows the evolution of normalized error values over time for the 3 groups. ANOVA_RM_ for the error indicated a significant main effect of time (F(9198) = 11.227, *p* < 0.001, ηp^2^ = 0.388), but no significant main effect of polarity (F(2,22) = 1.268, *p* = 0.301) or time × polarity interaction effect (F(18,198) = 1.022, *p* = 0.436). Post hoc analysis performed on the time main effect revealed only higher Error values for a-tDCS at B11 and B10 when compared to B3 and B11 when compared to B5 and for sham at B3 when compared to B12, B11, B10 and B9. P-level and effect size values are for a-tDCS B3 vs. B10 (*p* = 0.012, g = 1.33), B3 vs. B11 (*p* = 0.002, g = 1.30) and for B5 vs. B11 (*p* = 0.019, g = 1.06). P-level and effect size values are for sham B3 vs. B9 (*p* = 0.024, g = 1.05), B3 vs. B10 (*p* = 0.008, g = 1.12), B3 vs. B11 (*p* = 0.028, g = 1,03) and for B3 vs. B12 (*p* = 0.008, g = 1.09).

**Figure 5:**
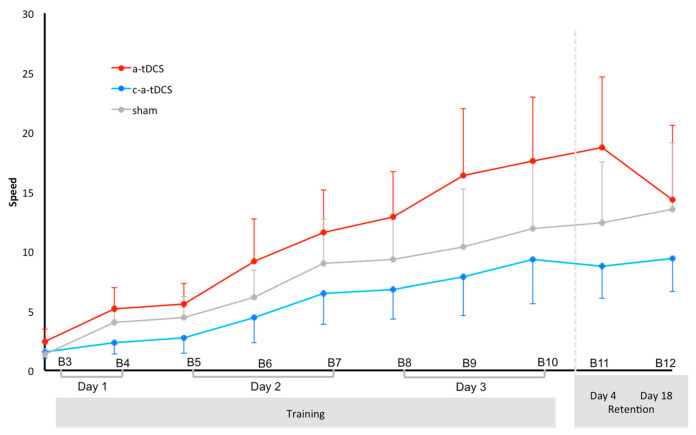
Evolution of of mean (and one SEM) speed over time (blocks—B) for each of the 3 groups. B3, B6 and B9 represent online tDCS. B3, B6 and B9 represent online tDCS. B4, B7 and B10 represent immediate offline. B5 and B8 represent delayed offline.

Figure 5 shows the evolution of normalized speed over time for the 3 groups. ANOVA_RM_ for the speed indicated a significant main effect of time (F(9198) = 13.966, *p* < 0.001, ηp^2^ = 0.338), but no significant main effect of polarity (F(2,22) = 0.830, *p* = 0.449) or time × polarity interaction (F(18,198) = 0.682, *p* = 0.826). Post hoc analysis performed on the time main effect revealed higher Speed values for a-tDCS at B11 and B10 when compared to B5, B4 and B3 and also for B3 when compared to B9 and B12; for c-a-tDCS at B12, B11 and B10 when compared to B5, B4 and B3 and also B9 when compared to B3; and for sham at B3 when compared to B12, B11 and B10. P-level and effect size values are for a-tDCS B3 vs. B9 (*p* = 0.004, g = 1.12), B3 vs. B10 (*p* = 0.001, g = 1.26), B3 vs. B11 (*p* < 0.001, g = 1.23), B3 vs. B12 (*p* = 0.029, g = 0.85), B4 vs. B10 (*p* = 0.017, g = 1.00), B4 vs. B10 (*p* = 0.005, g = 0.99) and for B5 vs. B10 (*p* = 0.027, g = 0.97), B5 vs. B11 (*p* = 0.009, g = 0.97). P-level and effect size values are for c-a-tDCS B3 vs. B9 (*p* = 0.013, g = 0.86), B3 vs. B10 (*p* < 0.001, g = 0.93), B3 vs. B11 (*p* = 0.002, g = 1.17), B3 vs. B12 (*p* < 0.001, g = 1.26), B4 vs. B10 (*p* = 0.003, g = 0.82), B4 vs. B11 (*p* = 0.01, g = 1.00), B4 vs. B12 (*p* = 0.003, g = 1.09), B5 vs. B10 (*p* = 0.007, g = 0.75), B5 vs. B11 (*p* = 0.021, g = 0.90) and B5 vs. B12 (*p* = 0.006, g = 0.98). P-level and effect size values are for sham B3 vs. B10 (*p* = 0.029, g = 0.90), B3 vs.B11 (*p* = 0.018, g = 1,07) and for B3 vs. B12 (*p* = 0.005, g = 1.07).

## 4. Discussion

The present study explored whether multiple sessions of cathodal priming and anodal tDCS (c-a-tDCS) over 3 consecutive days could further enhance motor learning and retention of an already learned visuo-motor task compared to a-tDCS or sham. The main findings of this study were that (i) multiple sessions of c-a-tDCS significantly further enhanced speed and IP above baseline training levels with a relatively minor decrease in accuracy over the 3 training days that were maintained over the 2 retention days, (ii) although the increase in IP was numerically greater for c-a-tDCS than a-tDCS or sham, a-tDCS showed a numerically greater increase in speed with concomitant reduced accuracy; while c-a-tDCS showed relatively stable accuracy with smaller increase in speed and (iii) these learning and retention performance changes for the real tDCS groups (c-a-tDCS and a-tDCS) were not significantly different from the sham group.

### 4.1. Influence of Cathodal Priming and Anodal tDCS on Motor Performance Retention

Our main findings with priming c-tDCS and a-tDCS are encouraging for inducing short and long-term retention. Motor performance retention was improved by adding priming c-tDCS to multiple sessions of atDCS and motor task training with a more persistent phenomenon (cf. Figure 3). These findings corroborated past results by Christova et al. [22] that reported priming c-tDCS (15 min) compared to sham a greater improvement in grooved pegboard task performance after learning with concurrent a-tDCS (1 mA, 20 min) for the non-dominant hand two weeks after. Similar to Christova et al. [22], we were not able to detect significant differences between groups 2 weeks after motor training. However, c-tDCS priming was found to be the unique condition with a significant difference from baseline with a large increase as indicated with the effect size (g = 2.87, Figure 3). In the present study, the motor performance gains 18 days after training were 22% with c-a-tDCS while a-tDCS priming and sham produced 3% and 20%, respectively. The willingness to combine priming c-tDCS and multiple sessions of a-tDCS and motor task training makes it difficult to account for the proportion of both factors in the final outcome. The lack of an experimental condition with sham priming followed by a-tDCS and motor-tracing task prevents concluding that the increase was due only to the c-tDCS priming effect. The need to have several training sessions seems to be a factor of first order, since Fujiyama et al. [23] reported for unimanual isometric force task that no significant difference between priming c-tDCS and sham persisted in a retest 24 h later.

A possible reason the sham group showed comparable learning and retention changes in circular task performance compared to the real tDCS groups may have been due to both (i) the effects of the sham group not reaching a plateau stage of learning compared to the real tDCS groups during the baseline training, and (ii) by the nature of using active sham, where there was 90 s of real stimulation applied during the training block. Therefore, we consider that the sham group in this study may not be an ideal control group to compare with the 2 tDCS groups. Nevertheless, compared to the c-a-tDCS and a-tDCS groups, the non-significant changes in performance (IP, accuracy, speed) for the sham group over the 2 baseline training blocks (B1 and B2) may have allowed greater potential for use dependent plasticity to show comparable learning and retention to that of the tDCS groups; while the 2 tDCS groups, that were working at the plateau stages of learning after the baseline training prior to tDCS training, adding tDCS to the training continued to enhance performance over the 3 days and more so for the c-a-tDCS group based on the speed–accuracy trade-off function (IP). Higher inhibitory tone at baseline, defined as a higher GABA/Glutamatergic metabolites ratio was shown to entail a greater disruptive effect of cathodal tDCS to response training gains [35]. Thus, the effects of cathodal and anodal tDCS on motor performance could be more beneficial when systems operate on suboptimal levels, e.g., regarding cortical excitability (not assessed in the present study) at baseline. However, the subtle differences in the changes in speed–accuracy trade-off (components of the IP) between c-a-tDCS (maintenance of accuracy over increasing speed) and a-tDCS (increasing speed over maintenance of accuracy) provides preliminary insights to a mechanistic modulation of performance with priming and polarity of tDCS.

The non-superiority of either priming c-tDCS or a-tDCS with motor training as compared to sham with motor training indicates that future studies are needed to control the intensity, the duration and the timing of application when manipulating priming. First, individualizing the tDCS intensity to ensure that excitability is lowered with cathodal tDCS is necessary [36]. Second, the duration of stimulation can play a role in modulating excitability since it was observed that 2 × 9 min without a break of c-tDCS with a conventional montage induced prolonged effects in cortical excitability changes compared to a single 9 min period [37]. However, a shorter duration of c-tDCS priming could be also more effective because an excessive and prolonged decrease in excitability may not lead to a return to the baseline during the 10 min of rest. In addition, using HD tDCS montage could induce delayed and longer lasting after-effects on motor cortex excitability as compared to conventional tDCS [13], suggesting further uncertainty in the timing of application. In our knowledge, no studies have been carried out to evaluate the optimal time to magnify the return to baseline with c-tDCS priming. Neuroimaging methods as electroencephalography or near infrared spectroscopy that can be combined with tDCS are a way to determine the optimal dosage [11,38]. Central to this endeavour is the definition of new biomarkers with such neuroimaging methods [11,39] to assess the effects of tDCS using a range of dosages in both research and translational (clinical) studies.

### 4.2. Impact of tDCS on a Low Learning Reserve Motor Task

The choice of a circular tracing-task with low learning reserve was voluntarily made to isolate the tDCS compared to the learning effects. While circular tracing-task based on the steering law derived from the Fitt’s law [40] should have provided limited improvement in performance despite training [41], an increase in IP and speed was revealed for the c-a-tDCS and a-tDCS group 24 h after the training without tDCS (B1 vs. B2), which indicates learning has improved performance; while no changes in these performance parameters were found for sham. This could suggest that both a-tDCS and c-a-tDS groups achieved already a plateau learning (ceiling levels) after the 2 first baseline blocks of training; while the sham did not. Beyond the will to propose a task to quickly reach the relative “ceiling” levels to be in line with highly skilled individuals (e.g., elite athletes, expert operators), the important use of the upper limbs in everyday life could disrupt the neuroplastic changes. Learning is so plastic that it is vulnerable to disruption by subsequent new learning [42]. Thus, to evaluate the interest to introduce priming, the replication of study without enhancement in performance after multiple training sessions is required with the addition of priming. For example, in a visuomotor grip force tracking task with stroke patients, no difference in the improvement in upper extremity Fugl–Meyer assessment at the end of 4 weeks training with concurrent a-tDCS was revealed compared to sham [43]. Beyond the heterogeneity of the degree of recovery for the patients and the weak intensity of stimulation (0.5 mA), the shoulder–elbow Fugl–Meyer assessment sub-score improved significantly more for a-tDCS compared to sham. This shows that a-tDCS combined with the chosen task allowed improvements in specific sub-components of clinical assessments. The addition of cathodal tDCS priming could potentially, for this disease, magnify the work of rehabilitation. Since stroke affects excitatory/inhibitory balance of the lesioned hemisphere towards greater inhibition, and the addition of a cathodal priming tDCS protocol to the lesioned hemisphere could further reduce excitability and allow a greater excitatory potential during the a-tDCS and arm rehabilitation training program, a greater potential to learn to use the arm again is expected.

### 4.3. Methodological Considerations

Despite the novelty of the current findings, some limitations should be highlighted to recommend caution in generalizing the results. The performance variability between subjects and groups could stem from differences in interpretation of the instruction set to perform the circular task by each subject, such as choosing a strategy with higher speed and accuracy being trade-off, and better accuracy with lower speed trade-off. This heterogeneity in the strategy each subject used to perform the task most likely led to the greater variability between subjects even though the IP metric tries to account for these different speed–accuracy strategies. Giving a more precise instruction to bias speed over accuracy should be pursued in future tDCS studies of motor cortex stimulation to focus on enhancing movement speed, since this region is primarily involved in encoding the speed of movements. In the present study, the greater increase in speed for the a-tDCS group who predominantly utilized a speed bias over accuracy at the outset had the most profound increases in the speed of movement over the 3 days of training. In a clinical application of tDCS for stroke rehabilitation, Hamoudi et al. [44] has shown that 5 consecutive days of visuo-motor pinch grip training with the addition of a-tDCS led to a predominantly speed-based shift in the speed–accuracy trade-off.

Owing to varying tDCS effects due to individual differences, personalized tDCS intervention should be customized and applied. In addition, in this exploratory study, a small sample per group was enrolled. It is thus definitely needed in future studies of a larger sample size to confirm and possibly expand the current findings. This study focused on only healthy participants; therefore, it is worthwhile to explore the effects of both cathodal and anodal tDCS on motor performance and learning in patients with diminished or impaired motor function wherein ceiling effects less emerge. Whilst this investigation employed behavioural and perceptual outcomes, whether tDCS elicited a neurophysiological effect remains uncertain.

## 5. Conclusions

This exploratory study showed that the motor performance changes observed with c-a-tDCS condition may hold promise for short and long-term retention of an already learned motor skill. However, the lack of significant difference for the c-a-tDCS condition compared to sham limits the current interpretation on the group level. Future powered studies of larger sample size are needed to optimize the instruction set and tDCS intensity, duration and the timing of priming application on the individual level. In addition, combined neurophysiological and neuroimaging techniques are required to fully understand the mechanism of action of the priming intervention at a larger scale and therefore confirm the interest of priming before concurrent anodal tDCS and motor task training on motor performance and retention.

## References

[1] Dissanayaka T., Zoghi M., Farrell M., Egan G.F., Jaberzadeh S. (2017). Does transcranial electrical stimulation enhance corticospinal excitability of the motor cortex in healthy individuals? A systematic review and meta-analysis. Eur. J. Neurosci..

[2] Saucedo Marquez C.M., Zhang X., Swinnen S.P., Meesen R., Wenderoth N. (2013). Task-specific effect of transcranial direct current stimulation on motor learning. Front. Hum. Neurosci..

[3] Schambra H.M., Abe M., Luckenbaugh D.A., Reis J., Krakauer J.W., Cohen L.G. (2011). Probing for hemispheric specialization for motor skill learning: A transcranial direct current stimulation study. J. Neurophysiol..

[4] Cuypers K., Leenus D.J.F., van den Berg F.E., Nitsche M.A., Thijs H., Wenderoth N., Meesen R.L.J., Kang E., Paik N., Reis J. (2013). Is Motor Learning Mediated by tDCS Intensity?. PLoS ONE.

[5] Reis J., Fischer J.T., Prichard G., Weiller C., Cohen L.G., Fritsch B. (2015). Time- but not sleep-dependent consolidation of tDCS-enhanced visuomotor skills. Cereb. Cortex.

[6] Besson P., Muthalib M., Dray G., Rothwell J., Perrey S. (2019). Concurrent anodal transcranial direct-current stimulation and motor task to influence sensorimotor cortex activation. Brain Res..

[7] Wiethoff S., Hamada M., Rothwell J.C. (2014). Variability in Response to Transcranial Direct Current Stimulation of the Motor Cortex. Brain Stimul..

[8] Li L.M., Uehara K., Hanakawa T. (2015). The contribution of interindividual factors to variability of response in transcranial direct current stimulation studies. Front. Cell. Neurosci..

[9] Cancelli A., Cottone C., Parazzini M., Fiocchi S., Truong D., Bikson M., Tecchio F. Transcranial Direct Current Stimulation: Personalizing the neuromodulation. Proceedings of the 2015 37th Annual International Conference of the IEEE Engineering in Medicine and Biology Society (EMBC).

[10] Edwards D., Cortes M., Datta A., Minhas P., Wassermann E.M., Bikson M. (2013). Physiological and modeling evidence for focal transcranial electrical brain stimulation in humans: A basis for high-definition tDCS. Neuroimage.

[11] Muthalib M., Besson P., Rothwell J., Perrey S. (2017). Focal Hemodynamic Responses in the Stimulated Hemisphere During High-Definition Transcranial Direct Current Stimulation. Neuromodulation Technol. Neural Interface.

[12] Datta A., Bansal V., Diaz J., Patel J., Reato D., Bikson M. (2009). Gyri-precise head model of transcranial direct current stimulation: Improved spatial focality using a ring electrode versus conventional rectangular pad. Brain Stimul..

[13] Kuo H.-I., Bikson M., Datta A., Minhas P., Paulus W., Kuo M.-F., Nitsche M.A. (2013). Comparing Cortical Plasticity Induced by Conventional and High-Definition 4 × 1 Ring tDCS: A Neurophysiological Study. Brain Stimul..

[14] Stagg C.J., Jayaram G., Pastor D., Kincses Z.T., Matthews P.M., Johansen-Berg H. (2011). Polarity and timing-dependent effects of transcranial direct current stimulation in explicit motor learning. Neuropsychologia.

[15] Cohen Kadosh R., Soskic S., Iuculano T., Kanai R., Walsh V. (2010). Modulating Neuronal Activity Produces Specific and Long-Lasting Changes in Numerical Competence. Curr. Biol..

[16] Schmidt R.A., Lee T.D. (2011). Motor Control and Learning: A Behavioral Emphasis.

[17] Ziemann U., Siebner H.R. (2008). Modifying motor learning through gating and homeostatic metaplasticity. Brain Stimul..

[18] Antal A., Nitsche M.A., Kincses T.Z., Kruse W., Hoffmann K.-P., Paulus W. (2004). Facilitation of visuo-motor learning by transcranial direct current stimulation of the motor and extrastriate visual areas in humans. Eur. J. Neurosci..

[19] Hashemirad F., Zoghi M., Fitzgerald P.B., Jaberzadeh S. (2016). The effect of anodal transcranial direct current stimulation on motor sequence learning in healthy individuals: A systematic review and meta-analysis. Brain Cognit..

[20] Karabanov A., Ziemann U., Hamada M., George M.S., Quartarone A., Classen J., Massimini M., Rothwell J., Siebner H.R. (2015). Consensus Paper: Probing Homeostatic Plasticity of Human Cortex With Non-invasive Transcranial Brain Stimulation. Brain Stimul..

[21] Nitsche M.A., Paulus W. (2001). Sustained excitability elevations induced by transcranial DC motor cortex stimulation in humans. Neurology.

[22] Christova M., Rafolt D., Gallasch E. (2015). Cumulative effects of anodal and priming cathodal tDCS on pegboard test performance and motor cortical excitability. Behav. Brain Res..

[23] Fujiyama H., Hinder M.R., Barzideh A., Van de Vijver C., Badache A.C., Manrique-C M.N., Reissig P., Zhang X., Levin O., Summers J.J. (2017). Preconditioning tDCS facilitates subsequent tDCS effect on skill acquisition in older adults. Neurobiol. Aging.

[24] Kumari N., Taylor D., Rashid U., Vandal A.C., Smith P.F., Signal N. (2020). Cerebellar transcranial direct current stimulation for learning a novel split-belt treadmill task: A randomised controlled trial. Sci Rep..

[25] Waters S., Wiestler T., Diedrichsen J. (2017). Cooperation Not Competition: Bihemispheric tDCS and fMRI Show Role for Ipsilateral Hemisphere in Motor Learning. J. Neurosci..

[26] Oldfield R.C. (1971). The assessment and analysis of handedness: The Edinburgh inventory. Neuropsychologia.

[27] Bikson M., Grossman P., Thomas C., Zannou A.L., Jiang J., Adnan T., Mourdoukoutas A.P., Kronberg G., Truong D., Boggio P. (2016). Safety of Transcranial Direct Current Stimulation: Evidence Based Update 2016. Brain Stimul..

[28] Gandiga P.C., Hummel F.C., Cohen L.G. (2006). Transcranial DC stimulation (tDCS): A tool for double-blind sham-controlled clinical studies in brain stimulation. Clin. Neurophysiol..

[29] Turski C.A., Kessler-Jones A., Hermann B., Hsu D., Jones J., Seeger S., Chappell R., Ikonomidou C. (2017). Feasibility and Dose Tolerability of High Definition Transcranial Direct Current Stimulation in healthy adults. Brain Stimul..

[30] Poreisz C., Boros K., Antal A., Paulus W. (2007). Safety aspects of transcranial direct current stimulation concerning healthy subjects and patients. Brain Res. Bull..

[31] Accot J., Zhai S. (2001). Scale effects in steering law tasks. Proceedings of the SIGCHI Conference on Human Factors in Computing Systems.

[32] Kulikov S., MacKenzie I.S., Stuerzlinger W. (2005). Measuring the effective parameters of steering motions. CHI ’05 Extended Abstracts on Human Factors in Computing Systems.

[33] Bonnetblanc F. (2008). Conflit vitesse-précision et loi de Fitts. Sci. Mot..

[34] Hedges L.V., Olkin I. (1985). Acknowledgments. Statistical Methods for Meta-Analysis.

[35] Filmer H.L., Ehrhardt S.E., Bollmann S., Mattingley J.B., Dux P.E. (2019). Accounting for individual differences in the response to tDCS with baseline levels of neurochemical excitability. Cortex.

[36] Berryhill M.E., Peterson D.J., Jones K.T., Stephens J.A. (2014). Hits and misses: Leveraging tDCS to advance cognitive research. Front. Psychol..

[37] Monte-Silva K., Kuo M.-F., Liebetanz D., Paulus W., Nitsche M.A. (2010). Shaping the Optimal Repetition Interval for Cathodal Transcranial Direct Current Stimulation (tDCS). J. Neurophysiol..

[38] Soekadar S.R., Herring J.D. (2016). Transcranial electric stimulation (tES) and NeuroImaging: The state-of-the-art, new insights and prospects in basic and clinical neuroscience. Neuroimage.

[39] Herold F., Gronwald T., Scholkmann F., Zohdi H., Wyser D., Müller N.G., Hamacher D. (2020). New Directions in Exercise Prescription: Is There a Role for Brain-Derived Parameters Obtained by Functional Near-Infrared Spectroscopy?. Brain Sci..

[40] Accot J., Zhai S. (1999). Performance evaluation of input devices in trajectory-based tasks. Proceedings of the SIGCHI Conference on Human factors in Computing Systems.

[41] Gibbs C.B. (1962). controller design: Interactions of controlling limbs, time-lags and gains in positional and velocity systems. Ergonomics.

[42] Shibata K., Sasaki Y., Bang J.W., Walsh E.G., Machizawa M.G., Tamaki M., Chang L.-H., Watanabe T. (2017). Overlearning hyperstabilizes a skill by rapidly making neurochemical processing inhibitory-dominant. Nat. Neurosci..

[43] Pavlova E.L., Lindberg P., Khan A., Ruschkowski S., Nitsche M.A., Borg J. (2017). Transcranial direct current stimulation combined with visuo-motor training as treatment for chronic stroke patients. Restor. Neurol. Neurosci..

[44] Hamoudi M., Schambra H.M., Fritsch B., Schoechlin-Marx A., Weiller C., Cohen L.G., Reis J. (2018). Transcranial Direct Current Stimulation Enhances Motor Skill Learning but Not Generalization in Chronic Stroke. Neurorehabilit. Neural Repair.

